# Endocan as a marker of endotheliitis in COVID-19 patients: modulation by veno-venous extracorporeal membrane oxygenation, arterial hypertension and previous treatment with renin–angiotensin–aldosterone system inhibitors

**DOI:** 10.1007/s00011-024-01964-8

**Published:** 2025-01-25

**Authors:** Marta Reina-Couto, David Alves, Carolina Silva-Pereira, Patrícia Pereira-Terra, Sandra Martins, João Bessa, Luísa Teixeira-Santos, Dora Pinho, Manuela Morato, Cláudia Camila Dias, António Sarmento, Margarida Tavares, João T. Guimarães, Roberto Roncon-Albuquerque, José-Artur Paiva, António Albino-Teixeira, Teresa Sousa

**Affiliations:** 1 Departamento de Biomedicina - Unidade de Farmacologia e Terapêutica, Faculdade de Medicina da Universidade do Porto (FMUP), Rua Dr. Plácido da Costa, S/N, Edifício Poente, Piso 3, 4200-450 Porto, Portugal; 2 Centro de Investigação Farmacológica e Inovação Medicamentosa da Universidade do Porto (MEDInUP), Alameda Prof. Hernâni Monteiro, 4200-319 Porto, Portugal; 3 Serviço de Medicina Intensiva, Centro Hospitalar e Universitário São João (CHUSJ), Alameda Prof. Hernâni Monteiro, 4200-319 Porto, Portugal; 4 Serviço de Farmacologia Clínica, CHUSJ, Alameda Prof. Hernâni Monteiro, 4200-319 Porto, Portugal; 5 Serviço de Patologia Clínica, CHUSJ, Alameda Prof. Hernâni Monteiro, 4200-319 Porto, Portugal; 6 Serviço de Nefrologia, Centro Hospitalar Universitário de Santo António, Largo Prof. Abel Salazar, 4099-001 Porto, Portugal; 7 iNOVA4Health, NOVA Medical School| Faculdade de Ciências Médicas, NMS|FCM, Universidade NOVA de Lisboa, Campo dos Mártires da Pátria 130, 1169-056 Lisboa, Portugal; 8 Departamento de Ciências do Medicamento, Laboratório de Farmacologia, Faculdade de Farmácia da Universidade do Porto, Rua Jorge Viterbo Ferreira nº 228, 4050-313 Porto, Portugal; 9 LAQV/REQUIMTE, Faculdade de Farmácia da Universidade do Porto, Rua Jorge Viterbo Ferreira nº 228, 4050-313 Porto, Portugal; 10 Departamento de Medicina da Comunidade, Informação e Decisão em Saúde, FMUP, Alameda Prof. Hernâni Monteiro, 4200-319 Porto, Portugal; 11 CINTESIS—Centro de Investigação em Tecnologias e Serviços de Saúde, Alameda Prof. Hernâni Monteiro, 4200-319 Porto, Portugal; 12 Serviço de Doenças Infecciosas, CHUSJ, Alameda Prof. Hernâni Monteiro, 4200-319 Porto, Portugal; 13 Departamento de Medicina, FMUP, Alameda Prof. Hernâni Monteiro, 4200-319 Porto, Portugal; 14 Unidade de Investigação em Epidemiologia (EPIUnit), Instituto de Saúde Pública da Universidade do Porto, Rua das Taipas 135, 4050-600 Porto, Portugal; 15 Departamento de Biomedicina– Unidade de Bioquímica, FMUP, Rua Dr. Plácido da Costa, S/N, Edifício Poente, Piso 2, 4200-450 Porto, Portugal; 16 Departamento de Cirurgia e Fisiologia, FMUP, Rua Dr. Plácido da Costa, S/N, Piso 6, 4200-450 Porto, Portugal

**Keywords:** COVID-19, Endocan, Endotheliitis, Hypertension, RAAS inhibitors, VV-ECMO

## Abstract

**Background and aims:**

Endocan has been scarcely explored in COVID-19, especially regarding its modulation by veno-venous extracorporeal membrane oxygenation (VV-ECMO), hypertension or previous renin–angiotensin–aldosterone system (RAAS) inhibitors treatment.

We compared endocan and other endotheliitis markers in hospitalized COVID-19 patients and assessed their modulation by VV-ECMO, hypertension and previous RAAS inhibitors treatment.

**Material and methods:**

Serum endocan, intercellular adhesion molecule-1 (ICAM-1), vascular cell adhesion molecule-1 (VCAM-1) and E-selectin were measured in “severe” (n = 27), “critically ill” (n = 17) and “critically ill on VV-ECMO” (n = 17) COVID-19 patients at admission, days 3–4, 5–8 and weekly thereafter, and in controls (n = 23) at a single time point.

**Results:**

Admission endocan and VCAM-1 were increased in all patients, but “critically ill on VV-ECMO” patients had higher endocan and E-Selectin. Endocan remained elevated throughout hospitalization in all groups. “Severe” and “critically ill” hypertensive patients or previously treated with RAAS inhibitors had higher endocan and/or VCAM-1, but in VV-ECMO patients the raised endocan values seemed unrelated with these factors. Among all COVID-19 hypertensive patients, those with previous RAAS inhibitors treatment had higher endocan.

**Conclusions:**

In our study, endocan stands out as the best marker of endotheliitis in hospitalized COVID-19 patients, being upregulated by VV-ECMO support, hypertension and previous RAAS inhibitor treatment.

**Supplementary Information:**

The online version contains supplementary material available at 10.1007/s00011-024-01964-8.

## Introduction

Since the outbreak of the COVID-19 pandemic caused by the severe acute respiratory syndrome coronavirus 2 (SARS-CoV-2) [1], many efforts have been made to elucidate its pathogenesis. Clinical manifestations of this novel disease range from asymptomatic to severe or critical progression requiring hospitalization and life-sustaining therapies such as mechanical ventilation or even, in selected cases, veno-venous extracorporeal membrane oxygenation (VV-ECMO) [2]. SARS-CoV-2 infects human cells by binding its spike protein to angiotensin converting enzyme-2 (ACE2), a membrane glycoprotein highly expressed in human epithelial cells of the lung and enterocytes of the small intestine [3, 4]. It is already well established that although SARS-CoV-2 has a greater tropism to infect pneumocytes, it has also the ability to trigger a multisystemic disease due to generalised vascular involvement [5, 6]. This hyperinflammatory and procoagulant state of COVID-19 implies the involvement of the endothelium, both as an effector and a target organ [7–9]. In fact, failure of the normal function of the endothelium, characterized not only by an imbalance between endothelium-derived vasodilators and vasoconstrictors but also by endothelial cell activation and consequent leukocyte recruitment and adhesion to the vessel wall, converges to a vasoconstrictor, proinflammatory and prothrombotic status associated with worse prognosis in COVID-19. Furthermore, although there is still controversy regarding the ability of SARS-CoV-2 to directly infect the endothelium, it is possible that viral components per se also induce endothelial dysfunction [10]. Moreover, endothelial dysfunction appears to persist beyond the acute phase and to contribute to the long-term effects of the disease [11, 12].

The relation between arterial hypertension and COVID-19 as well as the impact of antihypertensive drugs, such as renin–angiotensin–aldosterone system (RAAS) blockers, on SARS-CoV-2 infection and disease severity persist as two debatable topics since the beginning of the pandemic [13, 14]. Indeed, besides being the receptor for SARS-CoV-2 virus, ACE2 is also a key enzyme of the RAAS counterregulatory axis, contributing to cardiovascular regulation [15]. Since chronic RAAS blocker treatment may be potentially associated to an upregulation of ACE2 expression, there was a concern of a higher risk of infection and/or a more severe course of the disease [16]. While it is well established that hypertension is associated with endothelial dysfunction [17], some studies have concluded that hypertension does not play an independent role in SARS-CoV-2 infection and COVID-19 progression [18, 19]. Furthermore, although there is evidence supporting the safety and even the protective role of the RAAS blockers during COVID-19, a recently published randomized clinical trial in critically ill patients with COVID-19 showed that initiation of treatment with an angiotensin converting enzyme (ACE) inhibitor or an angiotensin receptor blocker during hospitalization did not ameliorate, and likely worsened, clinical outcomes [20]. Therefore, the the role of hypertension and RAAS blockers in COVID-19 is still a matter of debate.

Endocan, a soluble dermatan sulphate proteoglycan mainly secreted by the activated endothelium, has recently emerged as a promising prognostic biomarker in a broad spectrum of endothelial dysfunction-related pathologies such as sepsis, acute respiratory distress syndrome (ARDS), arterial hypertension and heart failure [21, 22]. In COVID-19 patients, there is also some evidence that higher endocan concentrations are associated with adverse outcomes [23–25], although there is still some controversy in the post-dexamethasone era. Nevertheless, despite endocan’s potential usefulness for cardiovascular risk stratification, its relationship with arterial hypertension and RAAS blocker treatment in COVID-19 patients has not been studied yet. Additionally, another important question, but still poorly addressed, is the impact of VV-ECMO support on endothelial function, and consequently on endocan concentrations, in critical COVID-19 patients, since ECMO initiation is known to be associated with an inflammatory response that may cause or aggravate endothelial injury [26, 27].

Hence, the aim of our study was to evaluate and compare the profiles of endocan and other endothelial dysfunction biomarkers (ICAM-1, VCAM-1 and E-selectin) in hospitalized patients with different stages of COVID-19 severity, including critical COVID-19 on VV-ECMO patients. Importantly, we also assessed the contribution of arterial hypertension or previous RAAS blocker treatment to endotheliitis as well as endocan association with inflammation, cardiac injury and outcomes in hypertensives versus normotensive patients.

## Material and methods

### Study design and population

The present study is part of a larger research project (RESEARCH 4 COVID-19 grant, project 519-reference number 613690173, “Unresolved inflammation and endothelitis in severe COVID-19 patients: identification of risk stratification biomarkers and therapeutic targets”, supported by FCT—Fundação para a Ciência e a Tecnologia, as part of a special call opened to fund rapid implementation projects for innovative response solutions to COVID-19 pandemic) involving patients from the ward of the Service of Infectious Diseases and from the ICUs of the Service of Intensive Care Medicine and the Service of Infectious Disease of a tertiary hospital (Centro Hospitalar Universitário São João, CHUSJ). Sixty-one patients (n = 61) with a laboratory-confirmed diagnosis of SARS-CoV-2 infection, defined by a positive result on an RT-PCR assay of a specimen collected on a nasopharyngeal swab, who were hospitalized in the context of hypoxemic respiratory failure and symptomatic for > 1 day, were consecutively enrolled in this single-centre cohort study, from September 2020 to February 2021. Most patients were recruited within 72 h of a positive RT-PCR result. Patients were excluded if they were under 18 years of age, were pregnant or lactating or had a history of vasculitis or connective tissue disease. Admission to the ward or ICU and the time for intubation and mechanical ventilation or VV-ECMO was based on clinical judgement according to “leges artis”. Patients were divided into two groups according to COVID-19 disease severity [2]: patients with severe COVID-19 (n = 27) admitted to the ward and patients with critical COVID-19 (n = 34) admitted to the ICU. The group of patients with critical COVID-19 was further subdivided into two groups according to the use or not of VV-ECMO: critically ill COVID-19 patients on VV-ECMO (critical COVID-19 on VV-ECMO, n = 17) and critically ill COVID-19 patients without VV-ECMO support (critical COVID-19, n = 17). Severe COVID-19 was characterized by the presence of oxygen saturation < 90% on room air, signs of pneumonia or signs of severe respiratory distress. Critical disease was defined as patients presenting criteria for Acute Respiratory Distress Syndrome (ARDS), sepsis, septic shock, or other conditions that require life-sustaining therapies, according to the World Health Organization’s guidelines [2]. Due to the prospective nature of our sampling, we were able to capture a heterogenous population of ward patients and ICU patients. Controls (n = 23) were recruited among healthy blood donor volunteers from the Service of Immunohemotherapy of CHUSJ before the COVID-19 pandemic. All eligible patients provided written informed consent to participate in the study. For ICU patients unable to give consent, this was solicited to their next of kin, and these patients provided informed consent retrospectively, where possible. Blood donor volunteers provided oral informed consent. The study was conducted in accordance with the Guidelines for Good Clinical Practice and the 1975 Declaration of Helsinki after approval by the CHUSJ Health Ethics Committee [CES 75-16], with project amended specifically for inclusion of subjects with COVID-19, within the scope of a RESEARCH 4 COVID-19 grant from FCT.

### Clinical data and sample collection

Patients were followed during their stay in the ward or ICU by the medical team of the project. Data regarding clinical and relevant demographic parameters were assessed for each patient by the medical team and were further anonymously coded to the project database, along with routine laboratory data, guaranteeing confidentiality. Illness severity was assessed by the Acute Physiology and Chronic Health Evaluation II (APACHE II) and Simplified Acute Physiology Score II (SAPS II) scoring systems at ICU admission. ICU length of stay, total hospital length of stay and mortality within 30 days and 1 year were also evaluated. The group of patients with critical COVID-19 on VV-ECMO included some patients who were previously hospitalized in the ICU of other hospitals before admission to the ICU of CHUSJ and that period was counted for the calculation of ICU length of stay. Also, all patient groups included a few patients that were further transferred from CHUSJ to other hospitals and all consecutive period of hospitalization was counted for calculation of total hospital length of stay.

For all patients, blood samples were collected at several time points throughout their hospital stay at CHUSJ, whenever possible: up to 48 h (days 1–2; admission), on days 3–4, on days 5–8 after admission and weekly thereafter until hospital discharge or a negative result in RT-PCR COVID-19 test. All collections of critical COVID-19 patients on VV-ECMO were started after VV-ECMO initiation. Blood samples from controls were collected at a single time point. All samples were processed within 1–2 h of collection and stored at -80ºC until assayed.

### Quantification of routine markers

All the routine laboratory analyses were performed at the Clinical Pathology Department of CHUSJ. Quantifications of lactate, partial pressure of oxygen (PaO_2_) and partial pressure of carbon dioxide (PaCO_2_) were performed by arterial blood gas analysis. Fraction of inspired oxygen (FiO_2_) was obtained from oxygen administration device and oxygen dose information in the medical records and the PaO_2_/FiO_2_ ratio was calculated. A Beckman Coulter® AU5800 automated clinical chemistry analyser (Beckman-Coulter, Hamburg, Germany) was used for the quantification of serum C-reactive protein (s-CRP) by an immunoturbidimetric assay and serum lactate dehydrogenase (s-LDH) by a spectrophotometric assay. Quantifications of plasma high-sensitivity troponin I (p-hsTnI), creatine kinase MB (p-CK-MB) and myoglobin (p-Myoglobin) were performed by chemiluminescent microparticle immunoassays using an Abbot® Architect i2000 automated analyser (Abbott® Diagnostics, Lake Forest, IL, USA).

### Quantification of endocan and other biomarkers of endothelial activation

Serum endocan (s-Endocan) was measured by an enzyme-linked immunosorbent assay (ELISA) using the commercial kit “Just Do It ELISA Kit H1” (JDIEK H1 assay, Lunginnov s.a.s, Lille, France). Other serum endothelium activation markers (serum intercellular adhesion molecule 1, s-ICAM-1; serum vascular cell adhesion molecule 1, s-VCAM-1; serum E-Selectin, s-E-Selectin) were evaluated by multiplex immunoassays using a Luminex 200 analyzer (Luminex Corporation, Austin, TX, USA), according to the protocols of Luminex Human Magnetic Assay (R&D Systems, Inc., Minneapolis, USA). Raw data analysis (mean fluorescence intensity) was performed using ISTM 2.3 software (Luminex Corporation).

### Quantification of proinflammatory biomarkers

Serum proinflammatory cytokines (serum tumor necrosis factor alpha, s-TNF-α; serum interleukin-1, s-IL-1β; serum interleukin-6, s-IL-6) were evaluated by multiplex immunoassays using with a Luminex 200 analyzer (Luminex Corporation, Austin, TX, USA), according to the protocols of MILLIPLEX® MAP Human High Sensitivity T Cell Magnetic Bead Panel (Millipore Corporation, Billerica, MA, USA). Raw data analysis (mean fluorescence intensity) was performed using ISTM 2.3 software (Luminex Corporation).

### Data and statistical analysis

Results are expressed as mean ± standard error of the mean (SEM) or as median (25th percentile; 75th percentile) for data with normal or non-normal distribution, respectively, or as percentage, and are graphically represented as Box and Whiskers plots. Statistical analysis was conducted using the GraphPad Prism 9 software (La Jolla, USA) and the IBM SPSS Statistics 27 software (IBM Corporation, New York, USA). Results were analysed by unpaired Student’s t-test or Mann–Whitney U-test, for comparisons between two groups, or by one-way ANOVA followed by a Tukey’s multiple comparison test or a Kruskal–Wallis test followed by a Dunn’s post hoc test, for comparison between three or more groups, where appropriate. Categorical variables were analysed by the Chi-Square or Fisher’s exact test. Biomarkers evolution throughout the hospitalization was analysed by Wilcoxon matched pairs signed rank test. Due to scarcity of samples at later time points, statistical analysis was only possible for results obtained until week 5 of hospitalization. We used Spearman’s correlation analysis to estimate correlations between sets of nonparametric data among all patients, hypertensive patients or normotensive patients. *P* values of < 0.050 were considered significant.

Repeated measures multivariate analyses were conducted to determine the relationship between s-Endocan (as the dependent variable) and some independent variables such as the COVID-19 patient group, hypertension, previous treatment with RAAS inhibitors or treatment with RAAS treatment along hospitalization, adjusted for age and gender, among all patients during the first week of hospitalization.

To prevent possible bias in clinical evaluation, all the patients were examined by the same medical team included in the project. To assure comparability of biomarkers assessment, samples from controls, severe COVID-19, critical COVID-19 and critical COVID-19 on VV-ECMO groups were evenly distributed in each assay plate. There were missing values in some biomarkers due to insufficient volume of samples or reagents to perform sample processing, dilution tests and assays. We had no permission to measure routine clinical biomarkers in controls (blood donor volunteers), or to access their hospital laboratory reports. The final number per group for the biomarkers/parameters evaluated at admission is shown in Suppl. Table 1. In addition, the number of patients decreased throughout hospitalization due to death, withdrawal of consent, hospital discharge or a negative RT-PCR COVID-19 test (Suppl. Figures 1–3). Moreover, there were some patients in whom it was not possible to collect blood samples in all the time points pre-specified in the study design throughout hospitalization due to medical/nurse team logistics, although they were maintained in the study as long as possible (Suppl. Figures 1–3). To avoid biasing the results, no imputation for missing values was used.

Sample size was defined according to the primary objectives of our FCT funded RESEARCH 4 COVID-19 project that consisted in characterizing resolution of inflammation and endotheliitis. Based on preliminary evaluations of endocan in healthy controls, patients with severe disease and critically ill patients, using power analysis, we calculated a sample size of 17 subjects per group to obtain an 80% power, at a 5% significance level (effect size-to-standard deviation ratio ca. 1). Since there was an elevated number of critically ill patients on VV-ECMO and a high heterogeneity of values between critically ill patients without VV-ECMO support versus those on VV-ECMO, we further divided the group of patients with critical COVID-19 into two groups: critically ill (without VV- ECMO) and critically ill on VV-ECMO. Despite this change, we had a total sample size of 84 subjects (i.e. more than 4 times the 17 initially estimated and with 17 patients per group in the two critically ill groups). Reporting of the study conforms to STROBE statement along with references to STROBE and the broader EQUATOR guidelines [28].

## Results

### Population demographic, clinical and biochemical characterization

Demographic, clinical and biochemical characteristics of the subjects included in the study are presented in Table 1.

**Table 1:** Demographic, clinical and biochemical characterization at admission and follow-up parameters of the study population

Demographic, Clinical and Biochemical parameters	Controls (n = 23)	Severe COVID-19 (n = 27)	Critical COVID-19 (n = 17)	Critical COVID-19 on VV-ECMO (n = 17)	*P* value
Age (Years)	**57 (53; 63)**	71 (63; 80)^**^	**67 (55; 72)**	**55 (40; 59)**^**###,$**^	** < 0.001**
Gender: Men, n (%)	15 (65)	17 (63)	11 (65)	11 (65)	0.999
Gender: Women, n (%)	8 (35)	10 (37)	6 (35)	6 (35)	0.999
Comorbidities, n (%)					
Diabetes	n.d	11 (41)	6 (35)	4 (24)	0.502
Obesity	n.d	7 (26)	8 (47)	10 (59)	0.081
Arterial Hypertension	n.d	18 (67)	13 (76)	8 (47)	0.188
Heart Failure	n.d	6 (22)	3 (18)	1 (6)	0.357
Respiratory Disease	n.d	8 (30)	4 (24)	2 (12)	0.389
Renal Disease	n.d	6 (22)	4 (24)	0 (0)	0.099
Malignancy	n.d	2 (7)	0 (0)	0 (0)	0.272
APACHE II Score	n/a	n/a	17 ± 2	19 ± 2	0.423
SAPS II Score	n/a	n/a	42 ± 4	40 ± 4	0.666
Previous Therapeutics, n (%)					
RAAS inhibitors prior to admission	**n/a**	**15 (56)**	**12 (71)**	**4 (24)**	**0.019**
Hypertensive patients on RAAS inhibitors prior to admission	**n/a**	**14 (52)**	**11 (65)**	**4 (24)**	**0.046**
Therapeutics at Admission, n (%)					
Dexamethasone	n/a	21 (78)	16 (94)	16 (94)	0.172
Remdesivir	n/a	1 (4)	0 (0)	2 (12)	0.263
Antibiotics	n/a	5 (19)	7 (41)	9 (53)	0.051
Vasopressor amines	**n/a**	**0 (0)**	**4 (24)**	**9 (53)**	** < 0.001**
PaO_2_/FiO_2_ ratio	n/a	**257 (230; 287)**	**92 (68; 137)**^**###**^	**100 (76; 119)**^**###**^	** < 0.001**
PaCO_2_ (mmHg)	n/a	**32 ± 1**	**37 ± 1**^**#**^	**48 ± 2**^**###, $$$**^	** < 0.001**
Lactate (mmol/L)	n/a	1.1 (1.0; 1.6)	1.5 (1.1; 1.8)	1.5 (1.2; 1.7)	0.258
Inflammatory Parameters					
s-TNF-α (pg/mL)	**11.4 (7.2; 15.1)**	19.9 (13.2; 31.5)^**^	26.3 (18.8; 37.3)^***^	21.8 (14.3; 30.0)^**^	** < 0.001**
s-IL-1β (pg/mL)	**0.3 (0.0; 0.7)**	**0.9 (0.3; 1.5)**	1.7 (1.2; 2.5)^**^	1.3 (0.8; 2.7)^**^	**0.001**
s-IL-6 (pg/mL)	**0.0 (0.0; 2.9)**	8.4 (5.2; 19.0)^***^	15.4 (5.7; 51.9)^***^	27.4 (4.1; 142.7)^***^	** < 0.001**
s-CRP (mg/L)	n.d	**100 (48; 173)**	**116 (78; 190)**	**163 (116; 245)**^**#**^	**0.016**
Cardiovascular Parameters					
SBP (mmHg)	n.d	124 (118; 135)	122 (106; 133)	119 (109; 127)	0.187
DBP (mmHg)	**n.d**	**75 (64; 88)**	**58 (55; 71)**^**##**^	**61 (57; 67)**^**##**^	** < 0.001**
p-hsTnI (ng/L)	n.d	12 (4; 2668)	6 (4; 22)	12 (4; 90)	0.573
p-CK-MB (ng/mL)	n.d	1.6 (0.3; 2.4)	1.1 (0.6; 1.6)	1.5 (0.9; 3.0)	0.544
p-Myoglobin (ng/mL)	n.d	191 (108; 645)	106 (63; 134)	86 (64; 241)	0.165
s-LDH (U/L)	n.d	349 (267; 453)	441 (333; 570)	558 (395; 573)	0.116
Follow-up					
Type of Oxygen Support During Hospitalization, n (%)					
Mechanical Ventilation	n/a	**2 (7)**	**11 (65)**	**17 (100)**	** < 0.001**
Non-invasive Ventilation	n/a	**5 (19)**	**11 (65)**	**14 (82)**	** < 0.001**
High-Flow Cannula	n/a	**9 (33)**	**13 (76)**	**9 (53)**	**0.020**
Supplementary Oxygen	n/a	26 (96)	13 (76)	16 (94)	0.081
ICU length of stay (days)	n/a	**0 (0; 0)**	**16 (7; 33)**^**###**^	**34 (16; 74)**^**###**^	** < 0.001**
Total Hospital length of stay (days)	n/a	**7 (5; 15)**	**22 (11; 57)**^**##**^	**43 (25; 116)**^**###**^	** < 0.001**
Mortality within 30 days, n (%)	n/a	3 (11)	4 (24)	1 (6)	0.287
Mortality within 1 year, n (%)	n/a	4 (15)	4 (24)	4 (24)	0.697

APACHE II, acute physiology and chronic health evaluation II; DBP, diastolic blood pressure; FiO_2_, fraction of inspired oxygen; ICU, Intensive Care Unit; n/a, not applicable; n.d., not determined; PaO_2_, partial pressure of arterial oxygen; PaCO_2_, partial pressure of carbon dioxide; p-CK-MB, plasma creatine kinase-MB; p-hsTnI, plasma high-sensitivity troponin I; SAPS II, Simplified Acute Physiology Score II; SBP, systolic blood pressure; s-CRP, serum C-reactive protein; s-IL-1β, serum interleukin 1 beta; s-IL-6, serum interleukin 6; s-LDH, serum lactate dehydrogenase; s-TNF-α, serum tumour necrosis factor alpha; VV-ECMO, veno-venous extracorporeal membrane oxygenation; Results are expressed as number (%), mean ± SEM or as median (25th percentile; 75th percentile) for data with normal or non-normal distribution, respectively. **P < 0.010 vs Controls; ***P < 0.001 vs Controls; ^#^*P* < 0.050 vs Severe; ^##^*P* < 0.010 vs Severe; ^###^*P* < 0.001 vs Severe; ^$^*P* < 0.050 vs Critical; ^$$$^*P* < 0.001 vs Critical. Bold values are shown for parameters with statistically significant differences between groups

Severe COVID-19 patients were significantly older than controls (*P* < 0.010), whilst critically ill COVID-19 on VV-ECMO patients were significantly younger than severe and critically ill COVID-19 patients (*P* < 0.001 and *P* < 0.050, respectively). There were no significant differences in gender between groups, but there was a predominance of males in all groups. Arterial hypertension was the most prevalent comorbidity in severe and critically ill COVID-19 patients, while obesity was the most prevalent in critical COVID-19 on VV-ECMO patients, although no significant differences were found between patient groups. There were no differences in APACHE II and SAPS II scores between the groups of critically ill patients.

There was a significant difference in the number of patients treated with RAAS inhibitors prior to admission (*P* = 0.019), with the critical on VV-ECMO patient group presenting a lower proportion of patients previously treated with these drugs (24%) compared to severe (56%) and critical (71%) groups. Furthermore, during hospitalization, RAAS treatment was maintained only in severe COVID-19 patients.

Regarding the therapeutics initiated at admission, almost all patients were treated with dexamethasone and very few were treated with remdesivir, with no differences between groups. There was a tendentially higher proportion of critically ill COVID-19 patients (with or without VV-ECMO) receiving antibiotics compared to severe patients (*P* = 0.051). Only critical COVID-19 groups had patients under vasopressor amine support (53% in Critical on VV-ECMO group and 25% in Critical group).

Lactate concentration at admission did not differ between patient groups, but PaCO_2_ was increased in critical patients, with higher values in VV-ECMO patients, and the PaO_2_/FiO_2_ ratio was significantly lower in both groups of critically ill patients compared to patients with severe COVID-19 (*P* < 0.001). Accordingly, there was a higher need for mechanical ventilation, non-invasive ventilation and high-flow cannula oxygen in all critical COVID-19 patients (with or without VV-ECMO) when compared with severe COVID-19 patients (*P* < 0.001, *P* < 0.001 and *P* = 0.020 respectively).

All COVID-19 patient groups presented significantly higher concentrations of inflammatory cytokines, such as s-TNF-α, s-IL-1β and s-IL-6, compared to controls. Additionally, s-CRP concentration was higher in patients with critical COVID-19 on VV-ECMO compared to severe COVID-19 patients (*P* < 0.050). Admission values of cardiovascular parameters did not differ between COVID-19 patient groups, except for diastolic blood pressure which was significantly lower in the critical groups than in severe patients (*P* < 0.001). Both groups of critically ill patients had a longer length of stay in the ICU than severe patients (*P* < 0.001), since only five severe COVID-19 patients needed a temporary upgrade of care to ICU in the first week of hospitalization [median ICU length of stay: 11 (3; 36) days]. At ICU admission, those five patients had mean APACHE II and SAPS II scores of 11 ± 2 and 28 ± 10, respectively. Both groups of critically ill patients had a longer total hospital length of stay when compared to severe COVID-19 patients (*P* < 0.001 and *P* < 0.010, respectively). Furthermore, the group of critical COVID-19 on VV-ECMO had a longer length of stay in ICU than the critical COVID-19 group, though not statistically significant. No significant differences in 30-day mortality nor in overall 1-year mortality were detected between COVID-19 patient groups. Additionally, the increase of mortality within 1-year was mostly observed in patients from the critical COVID-19 on VV-ECMO group.

### Endocan at admission and during hospitalization

**Fig. 1:**
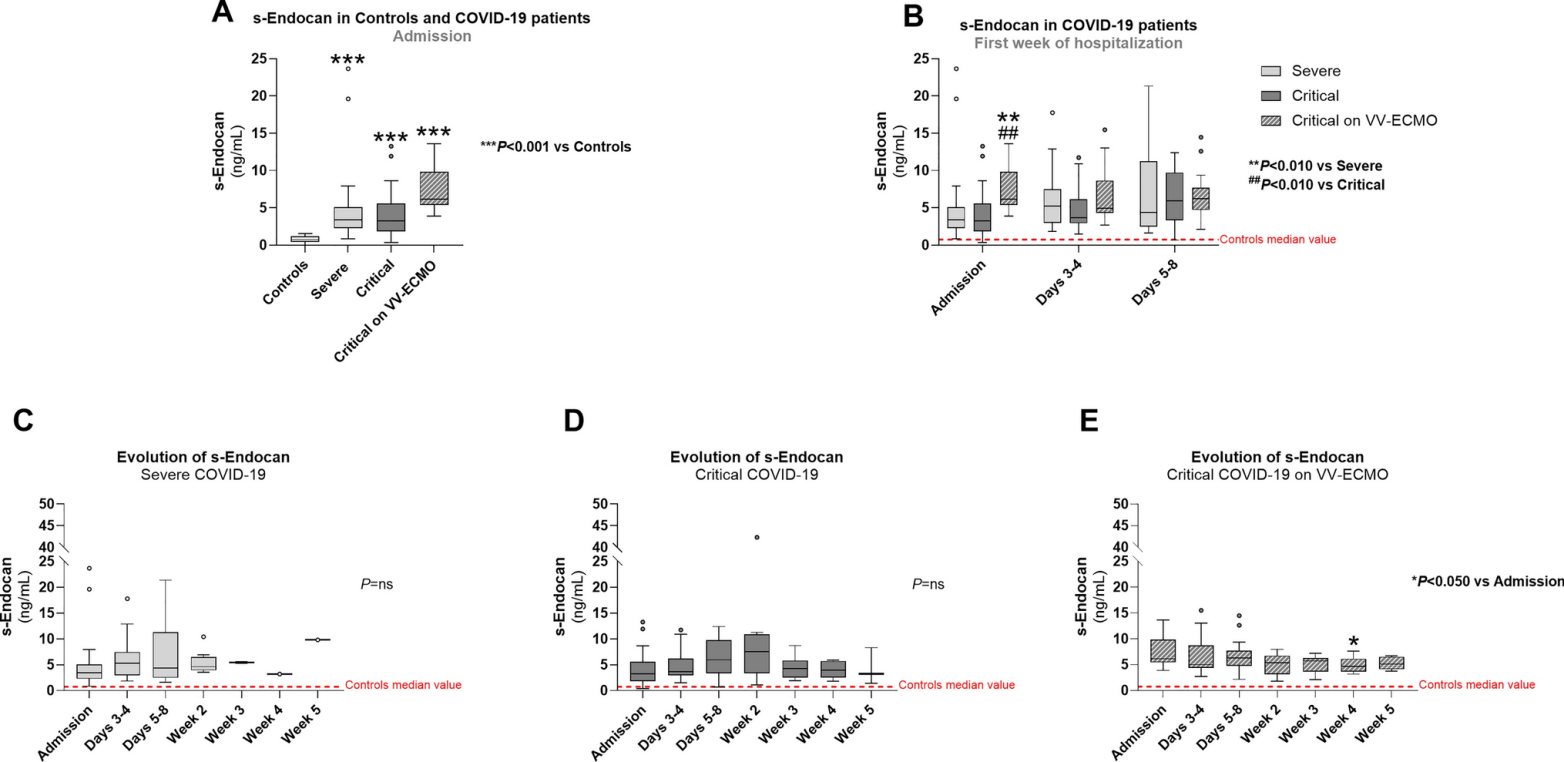
Serum endocan (s-Endocan) in all groups at admission (**A**), in COVID-19 patient groups during the first week of hospitalization (**B**) and throughout hospitalization in severe COVID-19 (**C**), critical COVID-19 (**D**) and critical COVID-19 on VV-ECMO (**E**). Results are presented in Box-and-Whiskers plot. s-Endocan, serum endocan; VV-ECMO, veno-venous extracorporeal membrane oxygenation

At admission, s-Endocan was significantly higher in all groups of COVID-19 patients, as compared to controls (*P* < 0.001) (Fig. 1A). When comparing only patient groups in the first week of hospitalization, we observed that critical COVID-19 on VV-ECMO group presented markedly higher admission concentration of s-Endocan than severe and critical COVID-19 groups (*P* < 0.010) (Fig. 1B), but no differences were found at days 3–4 and days 5–8 between patient groups (Fig. 1B).

During hospitalization, we found no significant alteration on s-Endocan values in patients with severe COVID-19 or critical COVID-19 (Fig. 1C and D). In patients with critical COVID-19 on VV-ECMO group, s-Endocan concentration was significantly reduced only at week 4 (*P* < 0.050 vs. Admission), although remaining quite above control values (Fig. 1E).

### Other biomarkers of endothelial activation at admission and during hospitalization

**Fig. 2:**
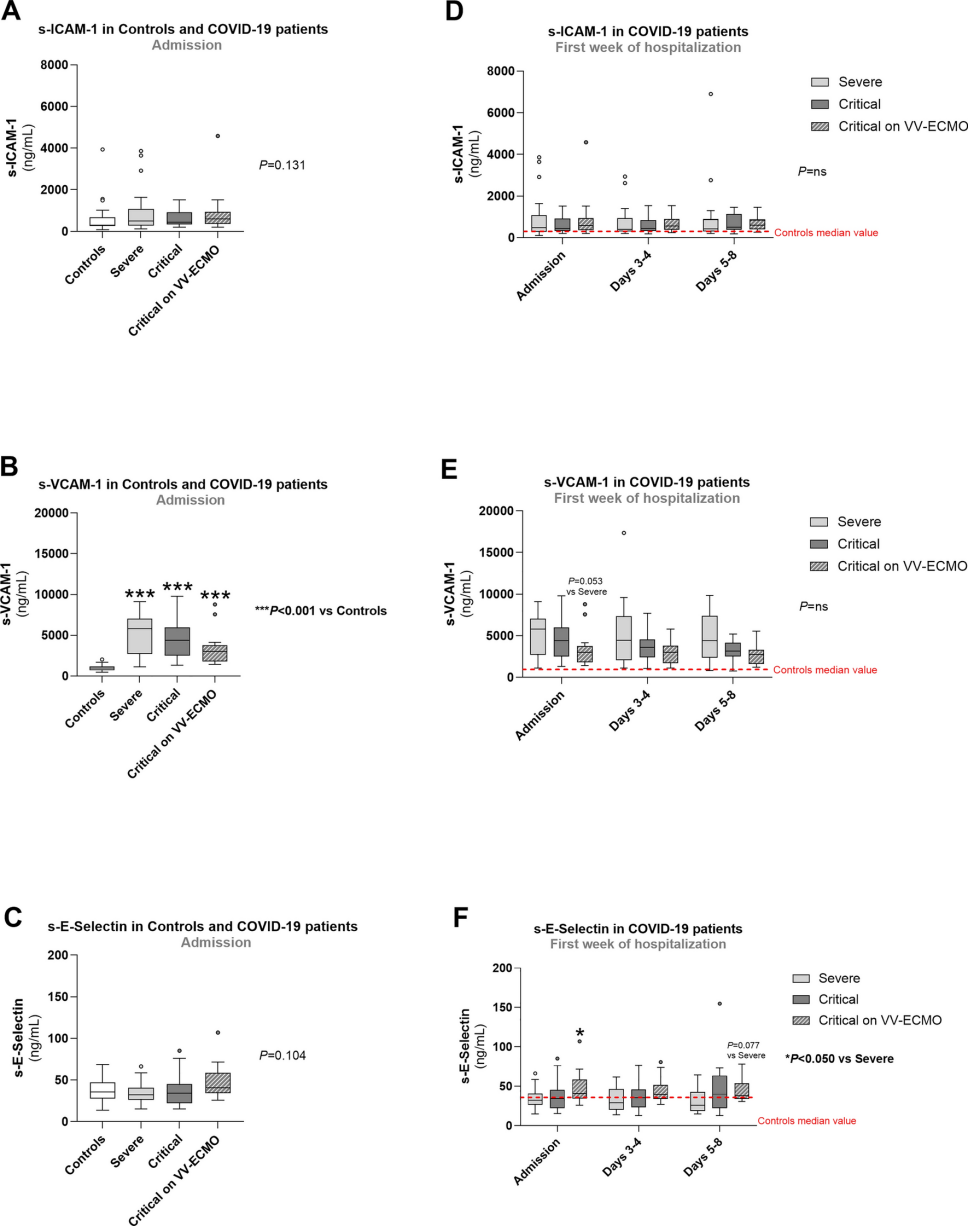
Comparison of serum endothelial activation markers (s-ICAM-1, s-VCAM-1, s-E-Selectin) in all groups at admission (**A**, **B** and **C**) and between COVID-19 patient groups during the first week of hospitalization (**D**, **E** and **F**). Results are presented in Box-and-Whiskers plot. s-ICAM-1, serum intercellular adhesion molecule 1; s-VCAM-1, serum vascular cell adhesion molecule 1; VV-ECMO, veno-venous extracorporeal membrane oxygenation

Concerning other endothelial activation biomarkers at admission, there were no significant differences regarding s-ICAM-1 and s-E-selectin concentrations when comparing all groups (Fig. 2A and Fig. 2C). However, all COVID-19 patient groups had significantly higher values of s-VCAM-1 compared to controls (*P* < 0.001) (Fig. 2B).

When comparing only patient groups in the first week of hospitalization, s-ICAM-1 and s-VCAM-1 values did not significantly differ between patient groups, although s-VCAM-1 values tended to be lower in patients with critical COVID-19 on VV-ECMO (*P* = 0.053 vs. severe, at admission) (Fig. 2D and E). Patients with critical COVID-19 on VV-ECMO also showed higher values of s-E-selectin compared to severe COVID-19 patients (*P* < 0.050 at admission and *P* = 0.077 at days 5–8) (Fig. 2F).

**Fig. 3:**
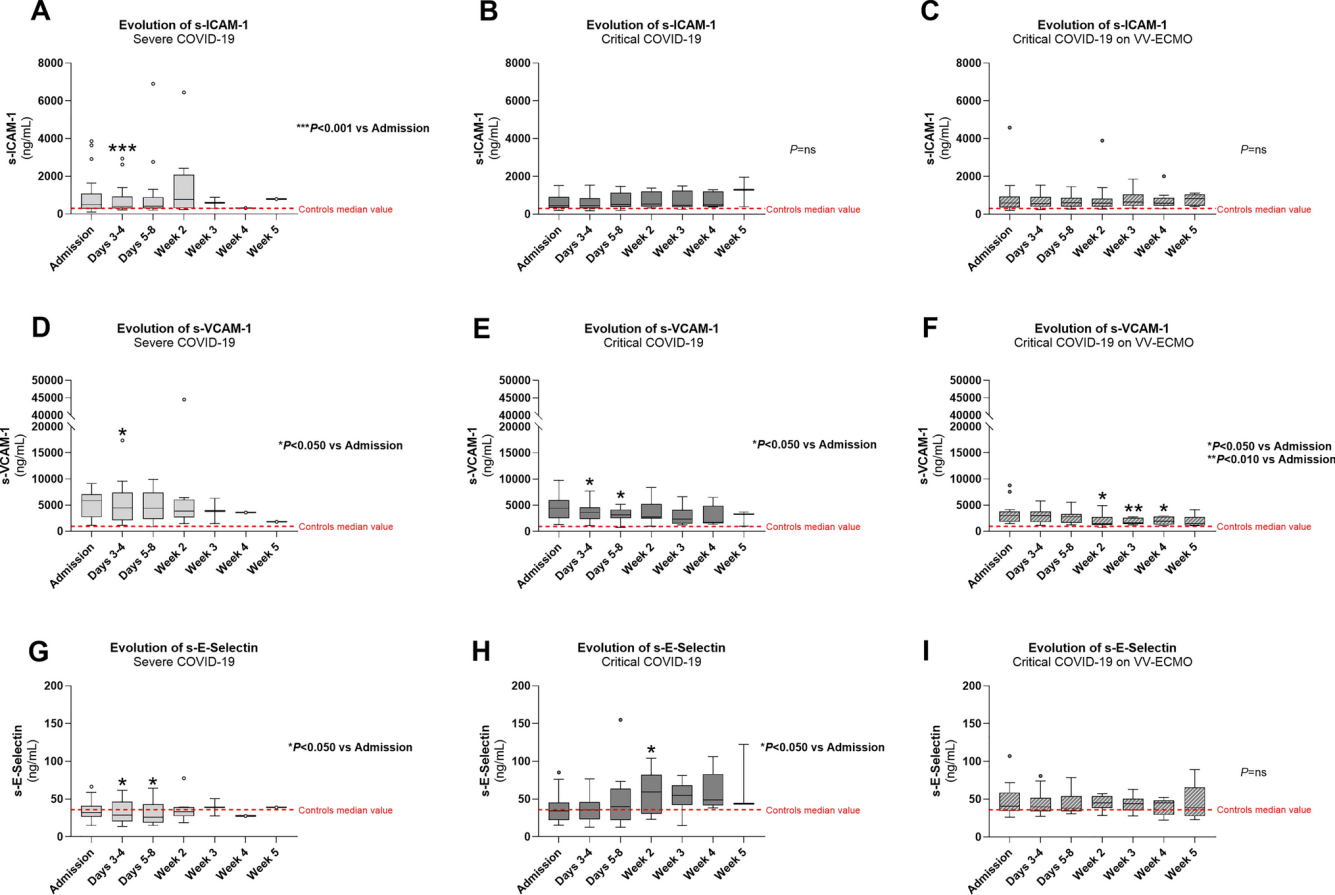
Serum endothelial activation markers (s-ICAM-1, s-VCAM-1, s-E-Selectin) profiles in patients with severe COVID-19 (**A**, **B** and **C**), critical COVID-19 (**D**, **E** and **F**) and critical COVID-19 on VV-ECMO (**G**, **H** and **I**) throughout hospitalization. Results are presented in Box-and-Whiskers plot. s-ICAM-1, serum intercellular adhesion molecule 1; s-VCAM-1, serum vascular cell adhesion molecule 1; VV-ECMO, veno-venous extracorporeal membrane oxygenation

During hospitalization, s-ICAM-1 concentrations only showed a significant reduction in severe COVID-19 patients at days 3–4 (*P* < 0.001 vs. Admission), remaining unchanged in both groups of critically ill patients (Fig. 3A–C). On the other hand, a decreasing pattern was observed for s-VCAM-1 values in all patient groups (Fig. 3D–F). Severe COVID-19 patients presented a significant reduction in s-VCAM-1 at days 3–4 (*P* < 0.050 vs. Admission) and the same happened for the group of critical COVID-19 at days 3–4 and days 5–8 (*P* < 0.050), but in critical COVID-19 on VV-ECMO patients, a significant reduction of s-VCAM-1 values was only observed at a later period, namely at weeks 2, 3 and 4 (*P* < 0.050, *P* < 0.010 and *P* < 0.050 vs. Admission, respectively). The concentration of s-E-Selectin was significantly reduced at days 3–4 and days 5–8 (*P* < 0.050 vs. Admission) in severe COVID-19 patients (Fig. 3G), but patients with critical COVID-19 showed a rising pattern in s-E-Selectin values, with a significant difference observed at week 2 (*P* < 0.050 vs. Admission) (Fig. 3H). There were no differences in s-E-Selectin concentrations during hospitalization in critical COVID-19 on VV-ECMO group (Fig. 3I).

### Impact of arterial hypertension or previous RAAS treatment on endocan and other biomarkers of endothelial activation during the first week of hospitalization

Arterial hypertension affected the values of s-Endocan, s-VCAM-1 and s-E-Selectin, but did not influence s-ICAM-1 concentration. Severe COVID-19 patients who were hypertensive presented markedly higher s-Endocan concentrations at days 3–4 and 5–8 (*P* = 0.026 and *P* = 0.008, respectively) (Table 2 and Suppl. Figure 4), as well as higher s-VCAM-1 values at days 3–4 and 5–8 (*P* = 0.044 and *P* = 0.003, respectively) compared to normotensives (Table 2). In the group of critical COVID-19 patients, the only marker affected by hypertension was s-VCAM-1, with significantly higher values being observed at admission and at days 3–4 (*P* = 0.010 and *P* = 0.045 vs. normotensive, respectively) (Table 2). Finally, s-E-selectin values were higher at days 5–8 in hypertensive patients from the critical COVID-19 on VV-ECMO group (*P* = 0.046 vs. normotensive) (Table 2). Of note, both normotensive and hypertensive critical COVID-19 on VV-ECMO patients presented similarly increased s-Endocan values throughout the first week of hospitalization, compared to controls and severe normotensive patients (Table 2 and Suppl. Figure 4).

**Table 2:** Impact of arterial hypertension or previous RAAS treatment on endocan and other biomarkers of endothelial activation during the first week of hospitalization

	Admission			Days 3–4			Days 5–8		
	Normotensive	Hypertensive	*P* value	Normotensive	Hypertensive	*P* value	Normotensive	Hypertensive	*P* value
Severe COVID-19									
s-Endocan (ng/mL)	2.6 (1.9; 4.4)	3.9 (2.4; 5.3)	0.231	**2.2 (2.0; 5.0)**	**6.1 (3.1; 9.8)**	**0.026**	**2.3 (2.0; 4.2)**	**8.6 (3.7; 12.1)**	**0.008**
s-ICAM-1 (ng/mL)	489 (310; 1238)	470 (272; 1150)	0.781	338 (258; 741)	406 (304; 1298)	0.391	387 (277; 2272)	416 (305; 1036)	0.875
s-VCAM-1 (ng/mL)	2780 (1353; 6559)	5822 (4427; 8433)	0.145	**2080 (1453; 4272)**	**5343 (2825; 8826)**	**0.044**	**1789 (1241; 2752)**	**5799 (4059; 9418)**	**0.003**
s-E-Selectin (ng/mL)	31.0 (26.4; 40.4)	32.4 (24.8; 41.9)	0.940	29.0 (22.0; 48.0)	29.0 (17.7; 46.6)	0.893	29.3 (20.6; 40.5)	25.5 (17.2; 46)	> 0.999
Critical COVID-19									
s-Endocan (ng/mL)	2.3 (0.7; 3.8)	4.2 (2.0; 7.1)	0.130	2.6 (1.6; 5.6)	3.7 (3.2; 6.5)	0.245	5.6 (4.8; 6.4)	6.0 (3.1; 10.5)	0.923
s-ICAM-1 (ng/mL)	450 (393; 1024)	385 (292; 908)	0.624	597 (473; 957)	350 (299; 854)	0.202	909 (639; 1178)	463 (341; 1118)	0.352
s-VCAM-1 (ng/mL)	**1928 (1450; 3313)**	**5075 (2868; 6132)**	**0.010**	**2113 (1512; 2610)**	**4122 (2786; 4962)**	**0.045**	2277 (1992; 2562)	3291 (3010; 4460)	0.132
s-E-Selectin (ng/mL)	31.0 (22.7; 65.7)	36.8 (22.0; 45.3)	0.785	43.7 (36.4; 58.2)	30.3 (20.2; 40.9)	0.102	51.9 (39.3; 64.6)	38.9 (18.4; 58.0)	0.550
Critical COVID-19 on VV-ECMO									
s-Endocan (ng/mL)	6.1 (5.3; 9.9)	6.6 (5.5; 9.7)	0.815	5.2 (4.2; 10.5)	4.9 (4.8; 8.8)	0.963	6.2 (3.5; 10.3)	6.2 (5.2; 7.3)	0.910
s-ICAM-1 (ng/mL)	481 (355; 846)	638.9 (360.2; 986.1)	0.673	605 (349; 907)	528 (426; 906)	0.963	596 (304; 881)	635 (499; 847)	0.673
s-VCAM-1 (ng/mL)	3124 (1764; 3447)	2983 (2375; 6712)	0.481	2199 (1574; 3601)	3239 (2279; 4255)	0.236	1649 (1414; 3287)	2984 (2205; 3400)	0.200
s-E-Selectin (ng/mL)	36.2 (34.3; 46.0)	50.0 (34.4; 64.9)	0.370	39.7 (33.8; 47.8)	43.1 (29.1; 56.2)	0.963	**34.2 (32.0; 41.6)**	**43.4 (38.1; 63.3)**	**0.046**

	Admission			Days 3–4			Days 5–8		
	Without RAAS inhibitors prior to admission	With RAAS inhibitors prior to admission	*P* value	Without RAAS inhibitors prior to admission	With RAAS inhibitors prior to admission	*P* value	Without RAAS inhibitors prior to admission	With RAAS inhibitors prior to admission	*P* value
Severe COVID-19									
s-Endocan (ng/mL)	2.7 (2.0; 4.0)	4.3 (2.4; 6.0)	0.300	**3.1 (2.1; 4.9)**	**7.4 (4.4; 10.6)**	**0.012**	**2.7 (2.1; 3.9)**	**10.8 (4.0; 12.3)**	**0.016**
s-ICAM-1 (ng/mL)	696 (293; 1609)	419 (260; 576)	0.217	640 (309; 1242)	388 (271; 928)	0.657	450 (301; 729)	383 (297; 1125)	> 0.999
s-VCAM-1 (ng/mL)	4860 (1805; 6866)	5828 (4635; 8340)	0.373	3329 (1837; 4758)	5895 (2841; 8964)	0.109	**2718 (1441; 3524)**	**6944 (3568; 9457)**	**0.036**
s-E-Selectin (ng/mL)	31.7 (27.1; 41.7)	32.3 (20.0; 40.9)	0.516	29.2 (25.4; 46.4)	28.7 (17.4; 47.1)	0.545	24.1 (23.1; 37.6)	27.4 (16.1; 47.4)	0.758
Critical COVID-19									
s-Endocan (ng/mL)	2.8 (0.9; 4.9)	3.7 (1.9; 7.9)	0.279	3.3 (1.6; 4.9)	3.8 (3.2; 6.8)	0.195	3.9 (1.2; 6.0)	7.4 (4.2; 11.1)	0.106
s-ICAM-1 (ng/mL)	459 (339; 1361)	381 (323; 661)	0.442	625 (311; 1202)	358 (315; 565)	0.442	909 (300; 1305)	463 (383; 1104)	0.540
s-VCAM-1 (ng/mL)	2361 (1695; 4797)	4782 (2860; 6143)	0.104	**1632 (1273; 3105)**	**4211 (2728; 5034)**	**0.009**	**2277 (1063; 2883)**	**3438 (3074; 4695)**	**0.008**
s-E-Selectin (ng/mL)	33.7 (23.8; 64.4)	35.6 (21.5; 44.4)	0.959	39.1 (25.3; 54.9)	32.2 (23.1; 42.2)	0.383	40.7 (19.2; 59.0)	38.9 (22.3; 65.8)	> 0.999
Critical COVID-19 on VV-ECMO									
s-Endocan (ng/mL)	6.1 (5.3; 9.9)	7.2 (5.7; 9.7)	0.624	4.9 (4.2; 8.7)	5.9 (4.9; 9.4)	0.412	5.4 (3.5; 7.6)	7.2 (5.6; 8.9)	0.464
s-ICAM-1 (ng/mL)	588 (365; 930)	508 (316; 3608)	0.785	605 (389; 928)	528 (379; 809)	0.703	607 (407; 912)	579 (400; 660)	0.624
s-VCAM-1 (ng/mL)	2941 (1809; 3447)	5297 (1950; 8482)	0.412	2898 (1671; 3795)	3239 (2132; 4213)	0.549	2389 (1522; 3306)	3082 (2359; 3355)	0.399
s-E-Selectin (ng/mL)	36.2 (32.7; 53.5)	54.5 (41.0; 70.6)	0.130	39.7 (33.8; 51.5)	43.1 (30.0; 67.6)	0.871	37.5 (32.9; 54.6)	43.4 (36.7; 57.8)	0.477

RAAS, renin–angiotensin–aldosterone system; s-ICAM-1, serum intercellular adhesion molecule 1; s-VCAM-1, serum vascular cell adhesion molecule 1; VV-ECMO, veno-venous extracorporeal membrane oxygenation. Results are expressed as median (25th percentile; 75th percentile). For each time point, bold values are shown for endothelial markers presenting statistically significant differences between normotensive and hypertensive patients or between patients with or without RAAS inhibitors prior to admission

Treatment with RAAS inhibitors prior to admission had a similar impact to that of hypertension on s-Endocan and s-VCAM-1. Among severe COVID-19 patients, those who were previously treated with RAAS inhibitors had markedly higher s-Endocan (Table 2 and Suppl. Figure 5) and s-VCAM-1 on days 3–4 and/or 5–8 than untreated patients (Table 2). Regarding critical COVID-19 group, prior treatment with RAAS inhibitors was also associated to significantly higher s-VCAM-1 values on days 3–4 and days 5–8. The concentration of s-Endocan was also higher on days 5–8, although not significantly, in critical COVID-19 patients previously treated with RAAS inhibitors (Table 2). In the critical on VV-ECMO group, no impact from previous treatment with RAAS inhibitors was detected in either s-Endocan or other endothelial biomarkers. However, critical on VV-ECMO patients consistently showed increased s-Endocan values during the first week of hospitalization, as compared to controls or untreated severe patients), independently of being or not previously treated with RAAS inhibitors (Table 2 and Suppl. Figure 5).

**Fig. 4:**
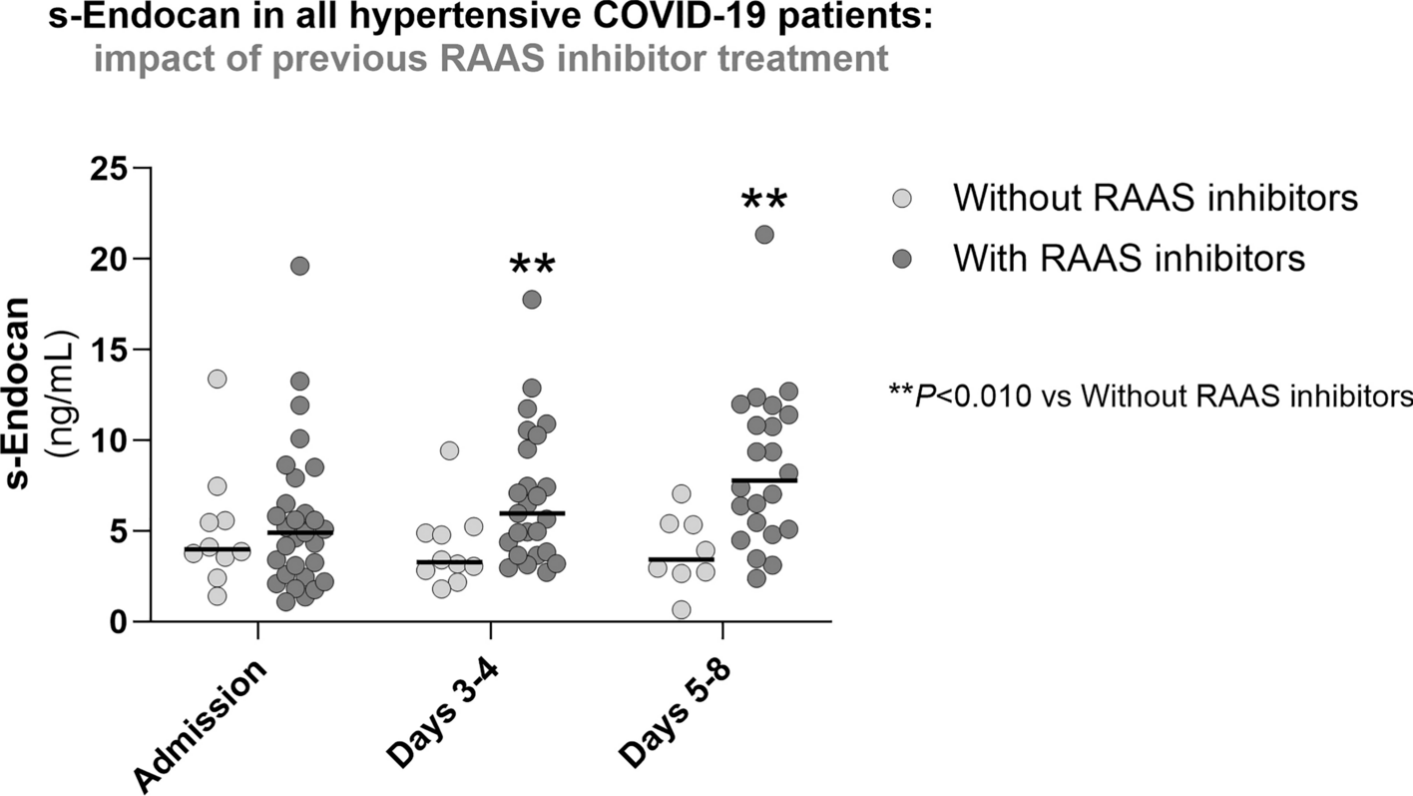
Impact of previous RAAS inhibitor treatment on serum endocan (s-Endocan) in all COVID-19 hypertensive patients during the first week of hospitalization. Results are expressed as scatter plots with median lines. RAAS, renin–angiotensin–aldosterone system; s-Endocan, serum endocan

In order to ascertain whether the increased values in s-Endocan or s-VCAM-1 were mainly due to hypertension or to previous treatment with RAAS inhibitors, we compared s-Endocan and s-VCAM-1 values in COVID-19 hypertensive patients previously treated with RAAS inhibitors and COVID-19 hypertensive patients not previously treated with RAAS inhibitors. Unexpectedly, we observed that hypertensive patients with prior RAAS inhibitors treatment had significantly higher s-Endocan concentration on days 3–4 and days 5–8 than hypertensive patients not previously treated with RAAS inhibitors (Fig. 4), but no differences were observed in s-VCAM-1 (data not shown). The increase in s-Endocan values was not due to a different proportion of use of other cardiovascular-related drugs such as diuretics, beta-blockers, calcium channel blockers, statins, antidiabetics or anticoagulants (Suppl. Table 2).

### Correlations of endocan in all patients, normotensive patients and hypertensive patients

Within all patients at admission, we observed significant positive correlations of s-Endocan with endothelial markers (s-VCAM-1, s-E-Selectin), proinflammatory cytokines (s-IL-6), PaCO_2_ and total hospital length of stay (Table 3).

**Table 3:** Correlations of S-Endocan at admission in all COVID-19 patients and separately in COVID-19 normotensive patients and in COVID-19 hypertensive patients

	S-Endocan (ng/mL)					
	All patients		Normotensive patients		Hypertensive patients	
	r Spearman	*P* value	r Spearman	*P* value	r Spearman	*P* value
APACHE II score	0.085	0.635	− 0.145	0.634	0.259	0.257
SAPS II score	− 0.040	0.824	− 0.462	0.114	0.218	0.342
PaO_2_/FiO_2_ ratio	− 0.020	0.889	0.230	0.358	− 0.145	0.405
PaCO_2_ (mmHg)	**0.458**	** < 0.001**	**0.483**	**0.023**	**0.429**	**0.008**
s-ICAM-1 (ng/mL)	0.243	0.059	0.237	0.289	0.261	0.109
s-VCAM-1 (ng/mL)	**0.281**	**0.028**	**0.458**	**0.032**	0.240	0.142
s-E-Selectin (ng/mL)	**0.399**	**0.001**	0.324	0.142	**0.468**	**0.003**
s-TNF-α (pg/mL)	0.001	0.992	0.021	0.927	− 0.029	0.860
s-IL-1β (pg/mL)	0.103	0.432	− 0.163	0.470	**0.324**	**0.044**
s-IL-6 (pg/mL)	**0.388**	**0.002**	0.347	0.114	**0.372**	**0.020**
s-CRP (mg/L)	− 0.113	0.384	− 0.278	0.210	0.005	0.978
SBP (mmHg)	0.078	0.548	0.040	0.859	0.095	0.566
DBP (mmHg)	− 0.079	0.457	− 0.051	0.822	− 0.084	0.611
p-hsTn I (ng/L)	0.217	0.278	− 0.326	0.254	**0.769**	**0.003**
p-CK-MB (ng/mL)	0.138	0.531	− 0.109	0.755	**0.635**	**0.030**
p-Myoglobin (ng/mL)	0.110	0.556	− 0.175	0.532	0.432	0.096
s-LDH (U/L)	0.257	0.120	− 0.168	0.604	**0.546**	**0.004**
ICU length of stay (days)	0.238	0.175	0.311	0.298	0.331	0.143
Total Hospital length of stay (days)	**0.431**	**0.001**	**0.579**	**0.005**	**0.365**	**0.023**

APACHE II, acute physiology and chronic health evaluation II; DBP, diastolic blood pressure; FiO_2_, fraction of inspired oxygen; ICU, Intensive Care Unit; PaO_2_, partial pressure of arterial oxygen; PaCO_2_, partial pressure of arterial carbon dioxide; p-CK-MB, plasma creatine kinase-MB; p-hsTnI, plasma high-sensitivity troponin I; SAPS II, Simplified Acute Physiology Score II; SBP, systolic blood pressure; s-CRP, serum C-reactive protein; s-ICAM-1, serum intercellular adhesion molecule 1; s-E-Selectin, serum E-selectin; s-IL-1β, serum interleukin 1 beta; s-IL-6, serum interleukin 6; s-LDH, serum lactate dehydrogenase; s-TNF-α, serum tumor necrosis factor alpha; s-VCAM-1, serum vascular cell adhesion molecule 1. Bold values are shown for parameters with statistically significant correlations

When considering only the normotensive patients, there were significant positive correlations of s-Endocan with s-VCAM-1, PaCO_2_ and total hospital length of stay, but within hypertensive patients, s-Endocan was significantly correlated with s-E-Selectin, proinflammatory cytokines (s-IL-1β, s-IL-6), biomarkers of cardiac injury (p-hsTnI, p-CK-MB, p-LDH), PaCO_2_ and total hospital length of stay (Table 3).

### Endocan during the first week of hospitalization in survivors versus non-survivors COVID-19 patients

The values of s-Endocan during the first week of hospitalization did not significantly differ between 30-day survivors and non-survivors COVID-19 patients (admission: survivors: 4.4 (2.5; 6.1) ng/mL versus non-survivors: 4.1 (2.0; 10.6) ng/mL, *P* = ns; days 3–4: survivors: 4.9 (3.2; 7.4) ng/mL vs non-survivors: 3.5 (2.9; 8.3) ng/mL), *P* = ns; days 5–8: survivors: 5.4 (3.2; 8.8) ng/mL vs non-survivors: 5.9 (3.0; 12.1) ng/mL, *P* = ns).

There were also no differences between s-Endocan values in the first week of hospitalization between 1-year survivors and non-survivors (admission: survivors: 4.2 (2.4; 6.1) ng/mL vs non-survivors: 5.4 (2.6; 7.2) ng/mL, *P* = ns; days 3–4: survivors: 5.0 (3.2; 7.5) ng/mL vs non-survivors: 3.7 (2.9; 6.0) ng/mL), *P* = ns; days 5–8: survivors: 5.9 (3.1; 9.4) ng/mL vs non-survivors: 5.4 (3.0; 7.8) ng/mL, *P* = ns).

### Repeated measures multivariate analysis

We further conducted repeated measures multivariate analysis in all patients during the first week of hospitalization to evaluate the association between s-Endocan (as the dependent variable) and independent variables, such as the group of patients (severe, critical or critical on VV-ECMO), hypertension, treatment with RAAS inhibitor prior to admission and treatment with RAAS inhibitor during hospitalization, adjusted for age and gender. We observed a significant inverse association between s-Endocan concentration and the lack of previous treatment with RAAS inhibitors (β = − 2.013; 95% CI: − 3.721; − 0.306; *P* = 0.021), meaning that patients not receiving RAAS inhibitors prior to hospitalization had lower values of s-Endocan than those receiving this treatment. There was also a borderline positive association between s-Endocan and critical on VV-ECMO group (β = 1.726; 95% CI = 0.926; 1138.614; *P* = 0.062) and a borderline inverse association between s-Endocan and the lack of hypertension (β = − 1.654; 95% CI: − 3.480; 0.173; *P* = 0.076). However, no association was found between s-Endocan values and treatment with RAAS inhibitor during hospitalization (β = 0.464; 95% CI: − 1.388; 2.315; *P* = 0.623).

## Discussion

Our study emphasizes endothelial dysfunction as a major feature in hospitalized COVID-19 patients and highlights s-Endocan as a putative biomarker. Importantly, we show that both arterial hypertension and previous treatment with RAAS inhibitors are associated with higher concentrations of s-Endocan and more intense endotheliitis. Also, our results suggest an unexpected deleterious impact of previous RAAS inhibitors treatment on endothelial function of hypertensive COVID-19 patients. Finally, s-Endocan positively correlated with proinflammatory cytokines and markers of cardiac injury only in hypertensive COVID-19 patients and with total hospital length of stay in both hypertensive and normotensive COVID-19 patients.

Previous studies have also shown increased endothelial dysfunction in COVID-19 patients vs controls, assessed by flow-mediated dilation or by systemic biomarkers of endothelial activation, [12, 24, 29–33]. Regarding endocan, a recent systematic review comprising a total of 686 participants concluded that endocan was markedly increased in COVID-19 patients and related to disease severity, with higher values in ICU patients and in non-survivors [34]. Furthermore, endocan was shown to be associated with COVID-19 complications, such as thrombotic events, need for oxygenation and acute respiratory failure [35]. In our study, we found raised values of endocan in all COVID-19 patients, with the highest admission values being observed in critical on VV-ECMO patients. Throughout hospitalization, endocan only showed a significant reduction in critical on VV-ECMO patients one month after admission, although remaining with concentrations quite above control values in all groups. This suggests that endothelial dysfunction is perpetuated for a long period in COVID-19 patients. In fact, recent studies confirmed that endothelial dysfunction persists many months after hospital discharge and is related to long COVID-19 symptoms [12, 36, 37], although endocan did not seem to be the best biomarker for this post-COVID-19 syndrome [38].

In a previous pilot study in COVID-19 patients on VV-ECMO, survival was associated with a marked decrease of endocan one week after VV-ECMO implantation [39]. In our patient cohort, we did not find a significant association of endocan with short- or long-term mortality. This was probably related to the use of dexamethasone in almost all patients, which might have contributed to the lower mortality rate observed, as stated by others [40, 41]. In fact, endocan was already shown to lose its prognostic ability after dexamethasone administration [29] and, apparently, the association between survival and the reduction of endocan concentration occurred only in COVID-19 on VV-ECMO patients not treated with dexamethasone [40]. Nevertheless, we observed that s-Endocan values at admission were positively correlated with total hospital length of stay, as well as with other endothelial activation markers (VCAM-1 and E-Selectin), proinflammatory status (IL-6) and lung gas exchange impairment (PaCO_2_). Indeed, endocan is known to be predominantly located in the pulmonary endothelium and associated with the inflammatory response, as evidenced by its induction under inflammatory conditions and its contribution to the upregulation of endothelial cell adhesion molecules [21, 42, 43]. In our cohort, at admission, the endothelial adhesion molecule s-VCAM-1 was also raised in all COVID-19 patient groups compared to controls, although without significant differences between severe and ICU groups, in line with a previous study showing similar admission VCAM-1 values for COVID-19 ICU and ward patients [44]. Additionally, we observed a reduction of s-VCAM-1 in all patient groups throughout hospitalization, which is in accordance with findings of comparable VCAM-1 concentrations in long COVID-19 patients and controls [44] if we assume that VCAM-1 returns to normal in some months. On the other hand, s-E-Selectin, another endothelial adhesion molecule, was higher in critical on VV-ECMO patients at admission, remaining elevated in this group and significantly increasing its concentration in the other critical COVID-19 group throughout hospitalization, which also corroborates the previously reported association of E-Selectin with severity and mortality in COVID-19 patients [31, 33]. Moreover, we observed that admission values of s-E-Selectin, but not s-VCAM-1, positively correlated with total hospital length of stay (data not shown), as also evidenced for s-Endocan. Thus, our results suggest that among the panel of endothelial markers measured, s-Endocan and s-E-Selectin are more directly related to disease severity, particularly with the need of VV-ECMO, for which there are no biochemical predictors so far.

There is a well-established relation between arterial hypertension and endothelial dysfunction, explained by various mechanisms such as RAAS activation, enhanced oxidative stress, inflammation and reduced NO bioavailability [18, 45]. Noteworthy, endocan has recently emerged as a promising biomarker in arterial hypertension, showing higher values in hypertensive patients than in normotensive individuals and a positive association with coronary artery disease among hypertensives [21, 46]. Similar findings have been found in clinical and experimental studies for E-selectin [47, 48], VCAM-1 [49, 50] or ICAM-1 [51, 52]. Arterial hypertension is a prevalent comorbidity in COVID-19 patients [13, 19] and a major risk factor for COVID-19 severity [53, 54], although the mechanisms predisposing to this association remain scarcely explored. Given that the endothelium is considered not only an effector but also a target in COVID-19, and that its dysfunction contributes to the multisystemic manifestations and poor outcomes of the disease [9], it is reasonable to assume that the underlying endothelial dysfunction in hypertensive patients potentiates the COVID-19 endotheliitis, which can be triggered directly by the SARS-CoV-2 virus or indirectly by the host systemic inflammatory response [19, 55]. Our results corroborate this hypothesis since hypertensive COVID-19 patients presented significantly higher s-Endocan and/or s-VCAM-1 concentrations than normotensives among severe and critical COVID-19 groups throughout the first week of hospitalization. Very few studies in COVID-19 patients studied the impact of hypertension on endothelial dysfunction biomarkers such as endocan, having concluded that hypertension was not associated with altered endocan concentrations in those patients [23, 35]. However, these studies only measured endocan at admission [35]. In our study, although s-Endocan did not differ between hypertensives and normotensives at admission, it was markedly increased in hypertensives on days 3–4 and days 5–8. Furthermore, hypertension was associated with a significant increase of s-VCAM-1 in critical patients and with higher s-E-Selectin in VV-ECMO patients. Interestingly, in critical on VV-ECMO patients, s-Endocan values were similarly high in both hypertensives and normotensives, indicating that other factors beyond hypertension contributed to the marked endotheliitis in these patients. Whether these increases reflect the degree of COVID-19 severity in these patients, or the VV-ECMO-induced inflammatory response and widespread endothelial activation, could only be ascertained if we had the opportunity to evaluate endothelial biomarkers during clinical deterioration, before VV-ECMO initiation, and to compare with other patients with similar severity and mechanical ventilation failure, with no indication to VV-ECMO. Given that no significant differences in APACHE II and SAPS II prognostic scores were found between our critical patients with or without VV-ECMO support, one could assume that the higher endothelial dysfunction in VV-ECMO patients resulted from endothelial derangements induced by the extracorporeal support, as suggested by others. [27]. However, these prognostic scores do not consider extracorporeal circuits, and neither PaO_2_/FiO_2_ nor PaCO_2_ were registered before ECMO cannulation, because many of these patients were rescued outside our hospital. Additionally, since we observed higher values of PaCO_2_ in the VV-ECMO group of patients, we cannot exclude the contribution of disease severity per se. In fact, this group included more patients with shock, as inferred by the higher use of vasopressor amines, and both shock status and catecholamine administration are known to be associated with exacerbated inflammation [56, 57]. Indeed, we observed that critical on VV-ECMO patients on vasopressor amines support had higher s-IL-6 and s-E-Selectin on days 5–8, although s-Endocan was not higher in these patients (data not shown).

Endocan has been shown to be associated not only with hypertension, but also with coronary artery disease [58, 59] and heart failure [22, 60]. In our present study, s-Endocan was positively correlated with markers of cardiac injury, such as hsTnI, CK-MB and LDH, and also with inflammatory cytokines, only among hypertensive patients, which highlights the contribution of hypertension and endocan to cardiovascular morbidity in COVID-19. Accordingly, a positive correlation between endocan and troponin I has also been evidenced in COVID-19 patients [61] and endotheliitis of small epicardial and intramyocardial vessels has been shown to be associated with myocardial injury in COVID-19 disease [62].

The negative impact of hypertension on COVID-19 severity could also be related to its treatment with RAAS blockers, which might potentially induce ACE2 upregulation and contribute to an increased risk of SARS-CoV-2 infection and a more severe disease course [19]. In the beginning of COVID-19 pandemic, these concerns have been mainly raised by preclinical studies showing an upregulation of ACE2 in cardiovascular and renal tissues of rats exposed to treatment with RAAS blockers [63, 64]. However, although the majority of clinical studies supports the safety of RAAS blockers treatment in COVID-19 patients [65–67], a recent randomized clinical trial in critically ill patients with COVID-19 showed that initiating treatment with an ACE inhibitor or an angiotensin receptor blocker during hospitalization did not improve, and likely worsened, clinical outcomes [20], but no mechanistic hypothesis was advanced. In our study, the analysis of endothelial biomarkers in patients previously treated or untreated with RAAS blockers evidenced a similar impact to that of arterial hypertension, with endocan and VCAM-1 being significantly higher in treated patients. However, when we further analysed the impact of previous treatment with RAAS blockers among hypertensive patients, significantly higher values of endocan on days 3–4 and on days 5–8 were evidenced in hypertensive patients previously treated with RAAS blockers. Of note, this was not due to a different proportion in the use of other cardiovascular-related drugs (e.g., antihypertensives, antidiabetics, statins and anticoagulants). Additionally, when we performed repeated measures multivariate analysis to identify variables affecting endocan values in COVID-19 patients, we only found a significant positive association with previous RAAS blockers treatment. Thus, our results suggest that prior treatment with RAAS blockers in hypertensive patients potentiates endothelial dysfunction in hospitalized COVID-19 patients. This could be due to the upregulation of RAAS components or RAAS escape mechanisms induced by previous treatment with RAAS blockers and potentiated by SARS-CoV-2 infection. So, the interactions between RAAS and COVID-19 are more complex than previously anticipated, having a significant impact on endothelitiis development in hypertensive patients.

This study has some limitations such as the small size of sample population and its single-center design. Moreover, we were not able to collect blood samples at all time points from all patients due to patient withdrawal of consent associated with the fear and psychological distress in hospitalized COVID-19 patients and also due to the high burden of clinical work during COVID-19 pandemic. Nevertheless, our study also has some strengths since we evaluated several endothelial dysfunction biomarkers in hospitalized COVID-19 patients with different disease severity, including the use of VV-ECMO, and were the first to explore the impact of arterial hypertension and previous RAAS treatment on endotheliitis throughout hospitalization.

Collectively, our results reinforce the intrinsic link between COVID-19 disease pathogenesis and the presence of endotheliitis, which persists throughout hospitalization and may contribute to the acute phase recovery and hospital length of stay in all groups. Also, endocan stands out as a major biomarker of endothelial derangement associated with VV-ECMO support in critical COVID-19 patients. Importantly and previously undescribed, arterial hypertension and prior treatment with RAAS blockers were shown to potentiate endotheliitis in hospitalized COVID-19 patients.

## Supplementary Information

Below is the link to the electronic supplementary material.

Supplementary file1 (TIF 112 kb)

Supplementary file2 (TIF 99 kb)

Supplementary file3 (TIF 100 kb)

Supplementary file4 (TIF 337 kb)

Supplementary file5 (TIF 357 kb)

Supplementary file6 (DOCX 15 kb)

Supplementary file7 (DOCX 16 kb)

Supplementary file8 (DOCX 13 kb)

## Data Availability

No datasets were generated or analysed during the current study.
