# Supplementary figures and images for: Endocan as a marker of endotheliitis in COVID-19 patients: modulation by veno-venous extracorporeal membrane oxygenation, arterial hypertension and previous treatment with renin–angiotensin–aldosterone system inhibitors
Source: Inflamm Res. 2025 Jan 25;74(1):26. doi: 10.1007/s00011-024-01964-8 (PMC11762693; doi:10.1007/s00011-024-01964-8)

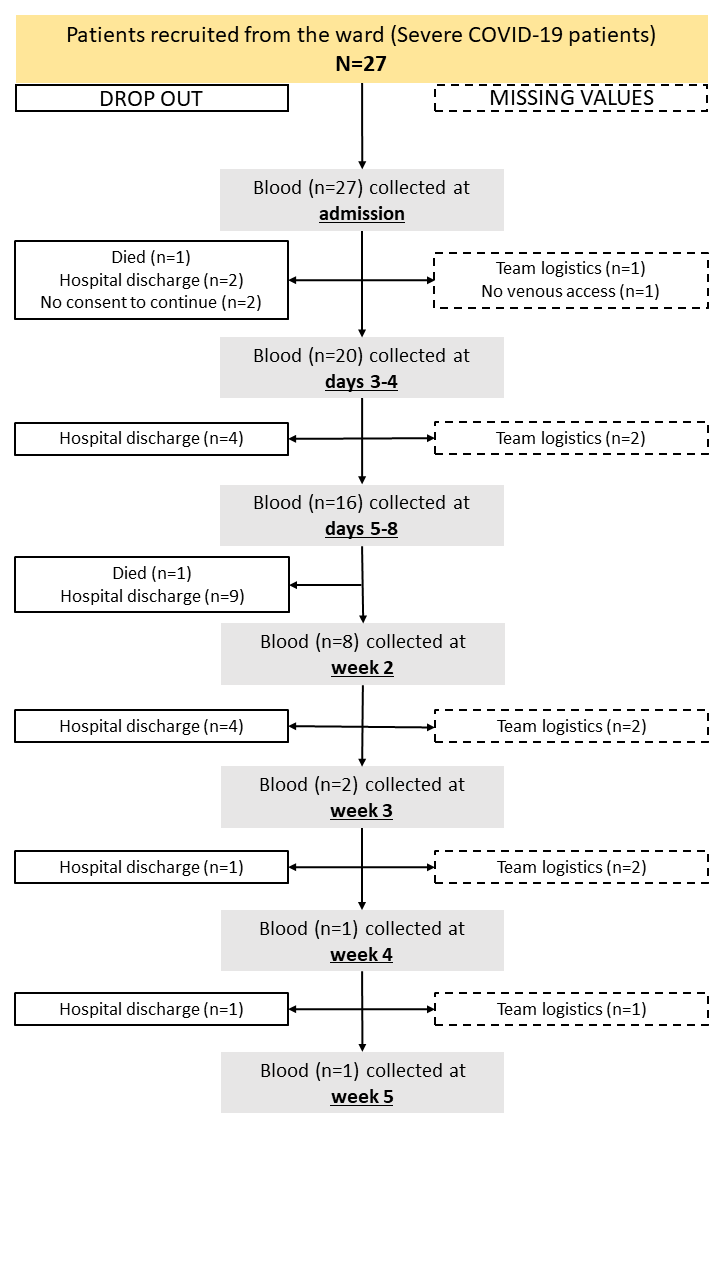

Supplement: Supplementary file 1 — Supplementary file1 (TIF 112 kb) [file 11_2024_1964_MOESM1_ESM.tif]

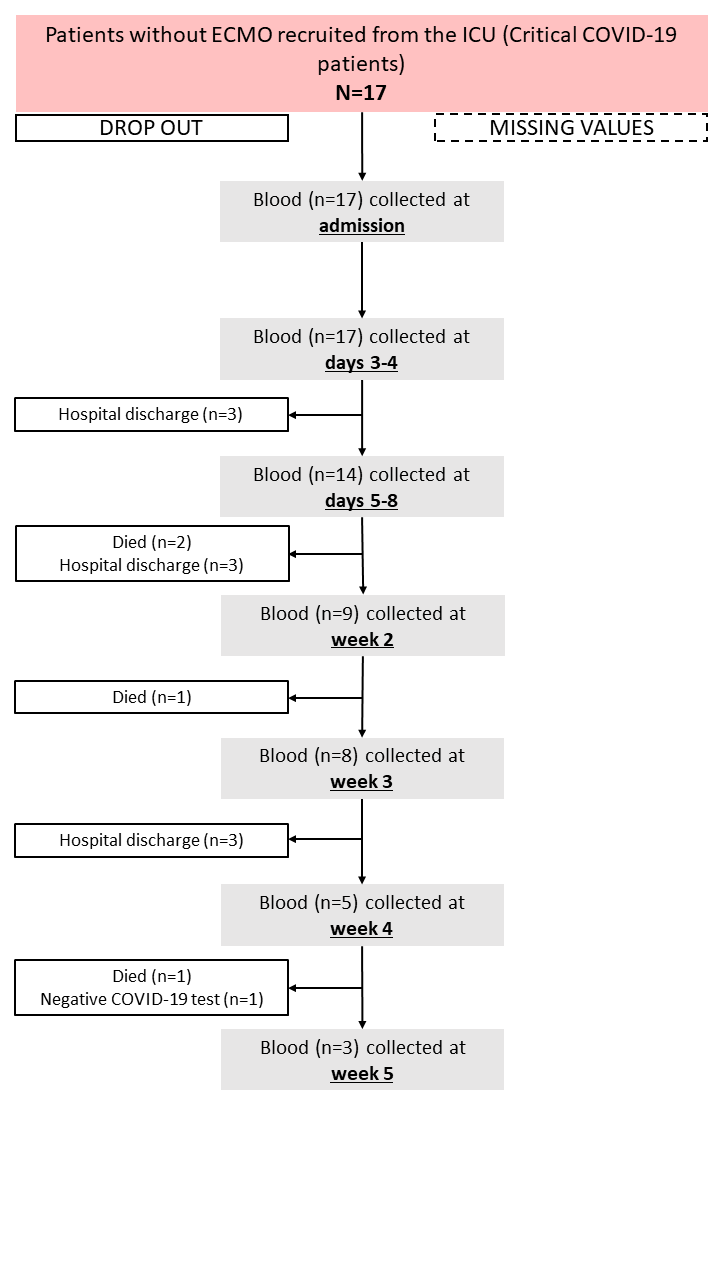

Supplement: Supplementary file 2 — Supplementary file2 (TIF 99 kb) [file 11_2024_1964_MOESM2_ESM.tif]

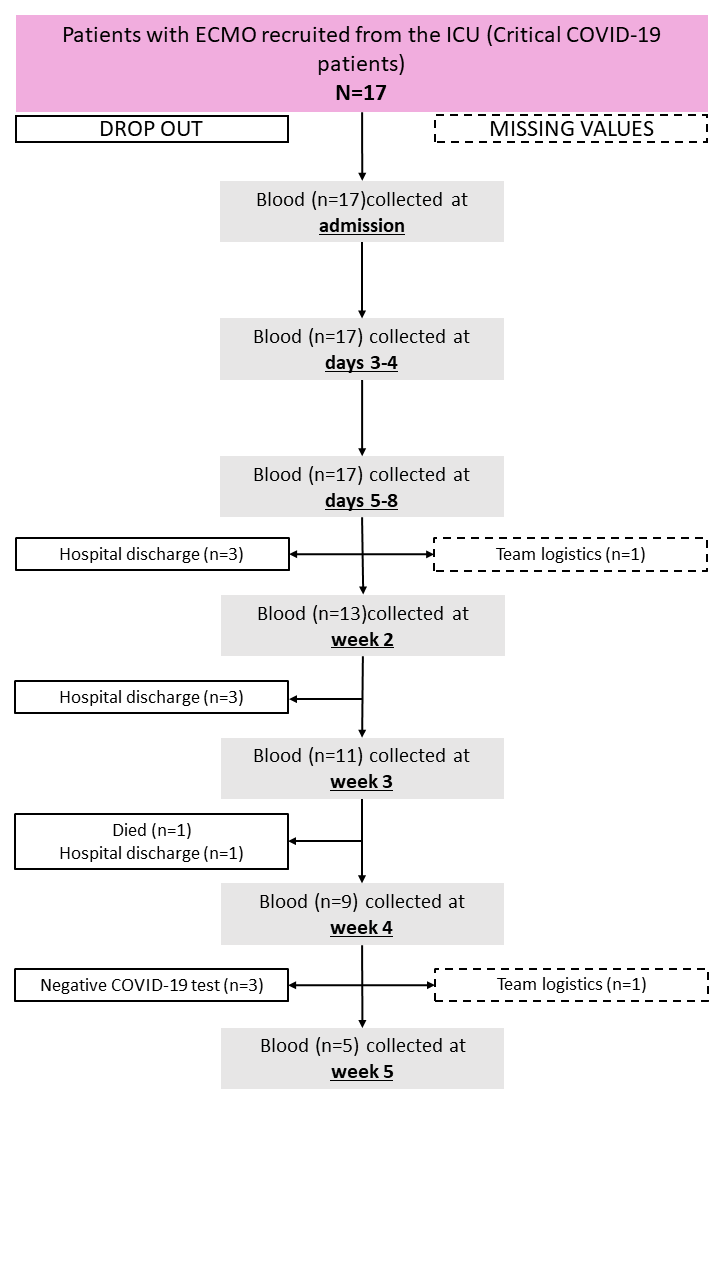

Supplement: Supplementary file 3 — Supplementary file3 (TIF 100 kb) [file 11_2024_1964_MOESM3_ESM.tif]

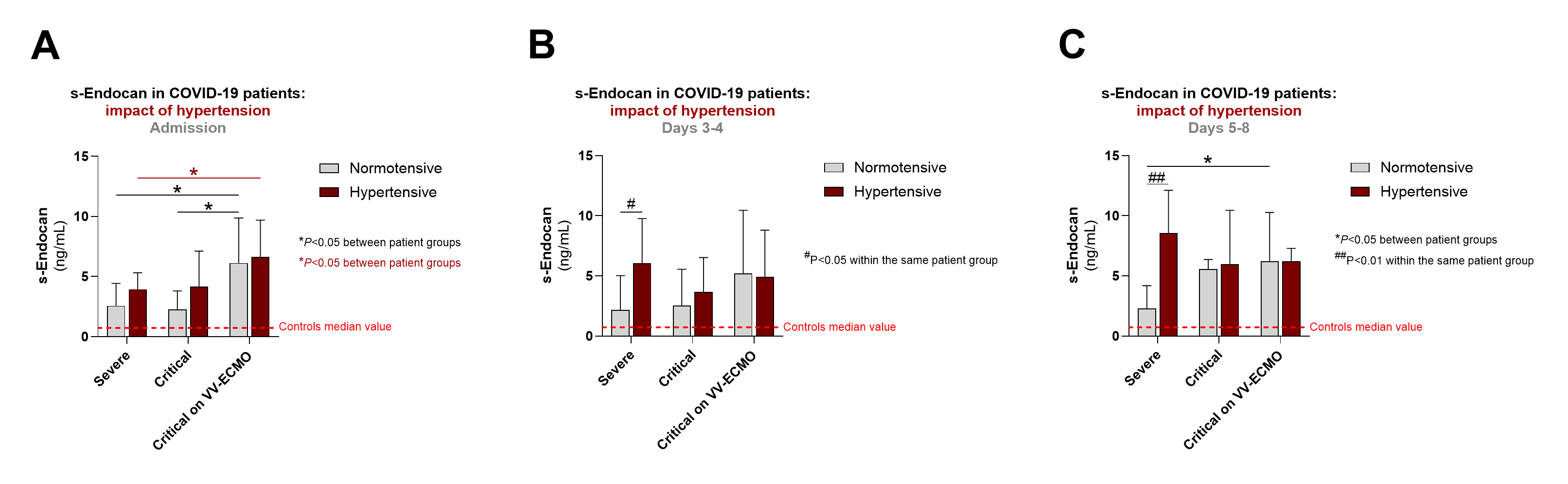

Supplement: Supplementary file 4 — Supplementary file4 (TIF 337 kb) [file 11_2024_1964_MOESM4_ESM.tif]

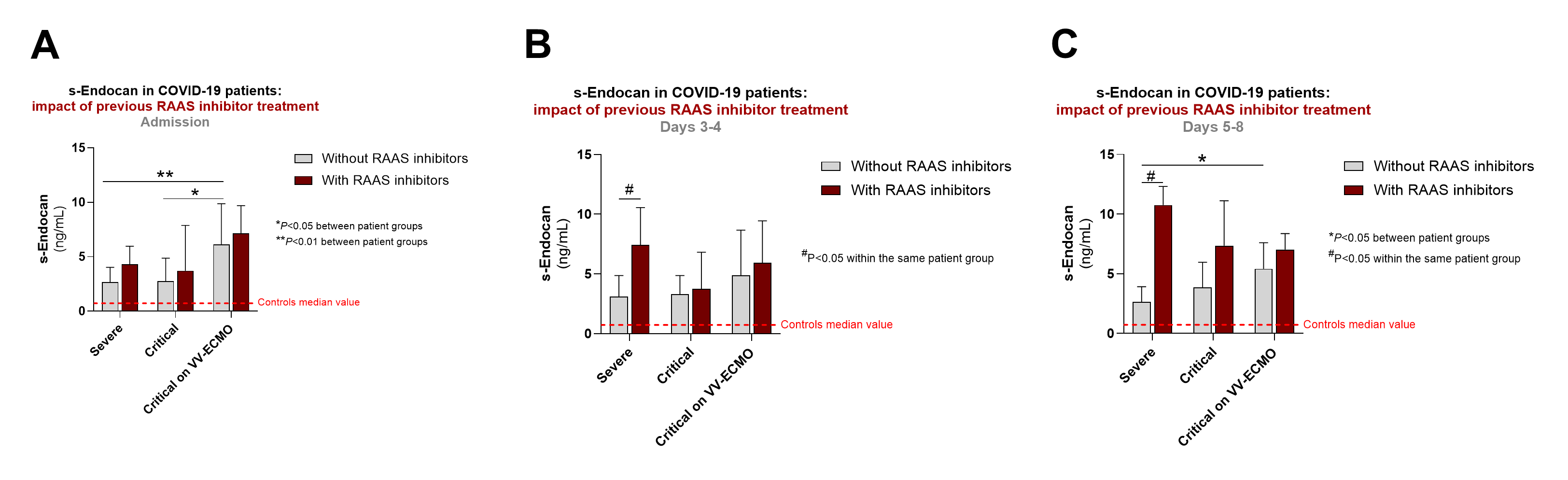

Supplement: Supplementary file 5 — Supplementary file5 (TIF 357 kb) [file 11_2024_1964_MOESM5_ESM.tif]
