# Supplementary material for: Endocan as a marker of endotheliitis in COVID-19 patients: modulation by veno-venous extracorporeal membrane oxygenation, arterial hypertension and previous treatment with renin–angiotensin–aldosterone system inhibitors
Source: Inflamm Res. 2025 Jan 25;74(1):26. doi: 10.1007/s00011-024-01964-8 (PMC11762693; doi:10.1007/s00011-024-01964-8)
Supplement: Supplementary file 6 — Supplementary file6 (DOCX 15 kb) [file 11_2024_1964_MOESM6_ESM.docx]

**Suppl. Fig. 1.** Flowchart indicating the number of severe COVID-19 patients analysed at each time point and the reasons for missing data and patient drop out from the study.

**Suppl. Fig. 2.** Flowchart indicating the number of critical COVID-19 patients analysed at each time point and the reasons for missing data and patient drop out from the study.

**Suppl. Fig. 3.** Flowchart indicating the number of critical COVID-19 on VV-ECMO patients analysed at each time point and the reasons for missing data and patient drop out from the study

**Suppl. Fig. 4.** Impact of hypertension on s-Endocan values in COVID-19 patients (within and between patient groups) during the first week of hospitalization: (A) Admission; (B) Days 3-4; (C) Days 5-8. VV-ECMO, veno-venous extracorporeal membrane oxygenation. Results are expressed as median with interquartile range. s-Endocan, serum endocan; VV-ECMO, veno-venous extracorporeal membrane oxygenation.

**Suppl. Fig. 5.** Impact of previous treatment with RAAS inhibitors on serum endocan values in COVID-19 patients (within and between patient groups) during the first week of hospitalization: (A) Admission; (B) Days 3-4; (C) Days 5-8. Results are expressed as median with interquartile range. s-Endocan, serum endocan; VV-ECMO, veno-venous extracorporeal membrane oxygenation.

**Suppl. Table 2.** Other drug treatments in hypertensive COVID-19 patients with or without previous treatment with RAAS inhibitors
