# Supplementary material for: Endocan as a marker of endotheliitis in COVID-19 patients: modulation by veno-venous extracorporeal membrane oxygenation, arterial hypertension and previous treatment with renin–angiotensin–aldosterone system inhibitors
Source: Inflamm Res. 2025 Jan 25;74(1):26. doi: 10.1007/s00011-024-01964-8 (PMC11762693; doi:10.1007/s00011-024-01964-8)
Supplement: Supplementary file 7 — Supplementary file7 (DOCX 16 kb) [file 11_2024_1964_MOESM7_ESM.docx]

**Suppl. Table 1.** Final number per group for each of the parameters at admission

| **Biomarkers/Parameters** | **Controls**  (n) | **Severe**  **COVID-19**  (n) | **Critical COVID-19**  (n) | **Critical COVID-19**  **on VV-ECMO**  (n) |
| --- | --- | --- | --- | --- |
| **APACHE II score** | **-** | **-** | 17 | 17 |
| **SAPS II score** | **-** | **-** | 17 | 17 |
| **PaO_2_/FiO_2_ ratio** | **-** | 25 | 17 | 11 |
| **PaCO_2_** (mmHg) | **-** | 25 | 17 | 17 |
| **Lactate** (mmol/L) | **-** | 21 | 15 | 16 |
| **Endothelial biomarkers** |  |  |  |  |
| **s-Endocan** (ng/mL) | 23 | 27 | 17 | 17 |
| **s-ICAM-1** (ng/mL) | 23 | 27 | 17 | 17 |
| **s-VCAM-1** (ng/mL) | 23 | 27 | 17 | 17 |
| **s-E-Selectin** (ng/mL) | 23 | 27 | 17 | 17 |
| **Inflammatory Parameters** |  |  |  |  |
| **s-TNF-α** (pg/mL) | 23 | 27 | 17 | 17 |
| **s-IL-1β** (pg/mL) | 23 | 27 | 17 | 17 |
| **s-IL-6** (pg/mL) | 22 | 27 | 17 | 17 |
| **s-CRP** (mg/L) | **-** | 27 | 17 | 17 |
| **Cardiovascular Parameters** |  |  |  |  |
| **SBP** (mmHg) | **-** | 21 | 12 | 4 |
| **DBP** (mmHg) | **-** | 21 | 12 | 4 |
| **p-hsTnI** (ng/L) | **-** | 5 | 10 | 12 |
| **p-CK-MB** (ng/mL) | **-** | 3 | 9 | 11 |
| **p-Myoglobin** (ng/mL) | **-** | 6 | 13 | 12 |
| **s-LDH** (U/L) | **-** | 25 | 8 | 5 |

APACHE II, acute physiology and chronic health evaluation II; DBP, diastolic blood pressure; FiO_2_, fraction of inspired oxygen; PaO_2_, partial pressure of arterial oxygen; PaCO_2_, partial pressure of carbon dioxide; p-CK-MB, plasma creatine kinase-MB; p-hsTnI, plasma high-sensitivity troponin I; SAPS II, Simplified Acute Physiology Score II; SBP, systolic blood pressure; s-CRP, serum C-reactive protein; s-IL-1β, serum interleukin 1 beta; s-IL-6, serum interleukin 6; s-LDH, serum lactate dehydrogenase; s-TNF-α, serum tumour necrosis factor alpha; VV-ECMO, veno-venous extracorporeal membrane oxygenation.
