# Supplementary material for: Endocan as a marker of endotheliitis in COVID-19 patients: modulation by veno-venous extracorporeal membrane oxygenation, arterial hypertension and previous treatment with renin–angiotensin–aldosterone system inhibitors
Source: Inflamm Res. 2025 Jan 25;74(1):26. doi: 10.1007/s00011-024-01964-8 (PMC11762693; doi:10.1007/s00011-024-01964-8)
Supplement: Supplementary file 8 — Supplementary file8 (DOCX 13 kb) [file 11_2024_1964_MOESM8_ESM.docx]

**Supplementary Table 2.** Other drug treatments in hypertensive COVID-19 patients with or without previous treatment with RAAS inhibitors

| **Other drugs, n (%)** | **Hypertensive COVID-19 patients without previous RAAS inhibitors treatment**  **(n=10)** | **Hypertensive COVID-19 patients with previous RAAS inhibitors treatment**  **(n=29)** | ***P*** |
| --- | --- | --- | --- |
| Diuretics | 3 (30.0%) | 15 (51.7%) | 0.290 |
| Beta blockers | 3 (30.0%) | 9 (31.1%) | >0.999 |
| Calcium channel blockers | 1 (10.0%) | 12 (41.4%) | 0.120 |
| Statins | 4 (40.0%) | 16 (55.2%) | 0.480 |
| Antidiabetics | 5 (50.0%) | 12.0 (41.4%) | 0.721 |
| Anticoagulants | 1 (10.0%) | 3.0 (10.3%) | >0.999 |

RAAS, renin-angiotensin-aldosterone system
